# A Review on the Effect of Traditional Chinese Medicine Against Anthracycline-Induced Cardiac Toxicity

**DOI:** 10.3389/fphar.2018.00444

**Published:** 2018-05-15

**Authors:** Xinyu Yang, Nian Liu, Xinye Li, Yihan Yang, Xiaofeng Wang, Linling Li, Le Jiang, Yonghong Gao, Hebin Tang, Yong Tang, Yanwei Xing, Hongcai Shang

**Affiliations:** ^1^Guang'anmen Hospital, Chinese Academy of Chinese Medical Sciences, Beijing, China; ^2^Key Laboratory of Chinese Internal Medicine of the Ministry of Education, Dongzhimen Hospital Affiliated to Beijing University of Chinese Medicine, Beijing, China; ^3^Department of Cardiology, Beijing Anzhen Hospital of the Capital University of Medical Sciences, Beijing, China; ^4^Department of Pharmacology, School of Pharmaceutical Sciences, South-Central University for Nationalities, Wuhan, China; ^5^Department of Pancreatic Oncology, Tianjin Medical University Cancer Institute and Hospital, Tianjin, China

**Keywords:** anthracyclines, traditional Chinese medicine, cardiac toxicity, adverse effects, antineoplastic

## Abstract

Anthracyclines are effective agents generally used to treat solid-tumor and hematologic malignancies. The use of anthracyclines for over 40 years has improved cancer survival statistics. Nevertheless, the clinical utility of anthracyclines is limited by its dose-dependent cardiotoxicity that adversely affects 10–30% of patients. Anthracycline-induced cardiotoxicity may be classified as acute/subacute or chronic/late toxicity and leads to devastating adverse effects resulting in poor quality of life, morbidity, and premature mortality. Traditional Chinese medicine has a history of over 2,000 years, involving both unique theories and substantial experience. Several studies have investigated the potential of natural products to decrease the cardiotoxic effects of chemotherapeutic agents on healthy cells, without negatively affecting their antineoplastic activity. This article discusses the mechanism of anthracycline-induced cardiotoxicity, and summarizes traditional Chinese medicine treatment for anthracycline-induced heart failure (HF), cardiac arrhythmia, cardiomyopathy, and myocardial ischemia in recent years, in order to provide a reference for the clinical prevention and treatment of cardiac toxicity.

## Introduction

Anthracyclines are cytostatic antibiotics that were discovered almost half a century ago (Salazar-Mendiguchia et al., 2014). Anthracycline agents are antibiotics isolated from the soil microbe *Streptomyces peucetius*, and are broad-spectrum, highly effective anti-cancer agents. Anthracyclines are usually used to treat solid-tumor and hematologic malignancies. The widespread use of anthracyclines for over 40 years has immensely improved cancer survival statistics (Geisberg and Sawyer, 2010). Nevertheless, the clinical utility of anthracyclines is limited by their dose-dependent cardiotoxicity which adversely affects 10–30% of patients (Vivenza et al., 2013). Several kinds of anticancer agents including anthracyclines, cyclophosphamide, trastuzumab, tyrosine kinase inhibitors, 5-fluorouracil, and angiogenesis inhibitors are associated with an increase in the risk of cardiovascular morbidity and mortality (Jain et al., 2017). Anthracycline-induced cardiotoxicity may be classified as acute/subacute or chronic/late toxicity (Appel et al., 2012). These types of toxicity lead to devastating adverse effects resulting in poor quality of life, morbidity, and premature mortality (Scott et al., 2011). For instance, the clinical use of anthracyclines is impeded by the development of increased QT duration, myocardial ischemia, arrhythmias (Saif et al., 2009), hypertension (Gressett and Shah, 2009), thromboembolic complications (Gressett and Shah, 2009), and myocardial dysfunction (Eschenhagen et al., 2011).

Traditional Chinese medicine has a long history of over 2,000 years, comprising several theories and rich experiences (Hao et al., 2015). Chinese herbal medicine is an essential part of traditional Chinese medicine. In China and other Asian countries, traditional Chinese medicine has been employed in clinical treatment for thousands of years (Zhang et al., 2016). Traditional Chinese medicine is increasingly welcomed in many developed countries, such as the United States and Australia. Several studies have investigated the potential of natural products in decreasing the cardiotoxic effects of chemotherapeutic agents on healthy cells, without negatively affecting their antineoplastic activity (Ohnishi and Takeda, 2015). Some medical workers turn to alternative or complementary treatments to prevent and treat anthracycline-induced cardiotoxicity. This article discusses the mechanism of anthracycline-induced cardiotoxicity, and summarizes traditional Chinese medicine treatment for anthracycline-induced HF, cardiac arrhythmia, cardiomyopathy, and myocardial ischemia in recent years, in order to provide a reference for the clinical prevention and treatment of cardiac toxicity.

## Mechanism of anthracyclines-induced cardiac toxicity

The mechanism of cardiotoxicity caused by chemotherapy drugs is very complex. It is the result of a multifactorial interaction which could lead to free radical damage, calcium overload, mitochondrial damage, apoptosis, iron metabolism disorder, and other mechanisms. These facilitate the occurrence and development of cardiac toxicity. Several mechanisms of anthracycline-induced cardiomyocyte injury have been proposed from studies involving animals and cell culture systems, but results from these studies are contradictory, suggesting a necessity for more studies (Jirkovsky et al., 2012). One potential mechanism of anthracycline-abducted cardiotoxicity involves the production of reactive oxygen species and site-specific DNA damage (Stěrba et al., 2013). Anthracyclines induce membrane damage through lipid peroxidation in organs, including the heart (Balanehru and Nagarajan, 1992). Although the formation of reactive oxygen species is induced by the quinone moiety of anthracyclines, oxidative stress can also be induced by nitric oxide synthase, resulting in the formation of nitric oxide (NO) and peroxynitrite (Fogli et al., 2004). Oxidative stress induction plays a primary role in anthracycline-induced cardiotoxicity (Vávrová et al., 2011) by inducing mitochondrial dysfunction, sarcomere damage, DNA damage, as well as a loss of pro-survival signaling (Force and Wang, 2013), which mediates both the survival and death of cardiomyocytes (Menna et al., 2010). The death of cardiomyocytes caused by both necrosis and apoptosis has also been implicated in anthracycline-induced cardiotoxicity, and the net loss of cells may further result in “cardiac wasting.” Another potential mechanism of anthracycline-induced cardiotoxicity involves the chelation reaction between iron and the α-ketol group of doxorubicin and epirubicin chemotherapeutic drugs (Eizaguirre et al., 2012). Furthermore, the preventive efficacy of deferoxamine, which is an iron chelator, also supports the hypothesis of iron involvement in anthracycline-induced cardiotoxicity (Cascales et al., 2012). Finally, anthracycline-induced changes in adenylate cyclase and adrenergic function (Calderone et al., 1991; Fu et al., 1994), together with abnormalities in Ca^2+^ handling (Takahashi et al., 1998), are all pivotal to the dynamic modulation of cardiac function. The extent of the contribution of each of these to the dose-dependent clinical HF in anthracycline-treated patients remains controversial.

## Protective effects of traditional chinese medicine and its constituent compounds on cardiotoxicity induced by anthracyclines

**Figure 1:**
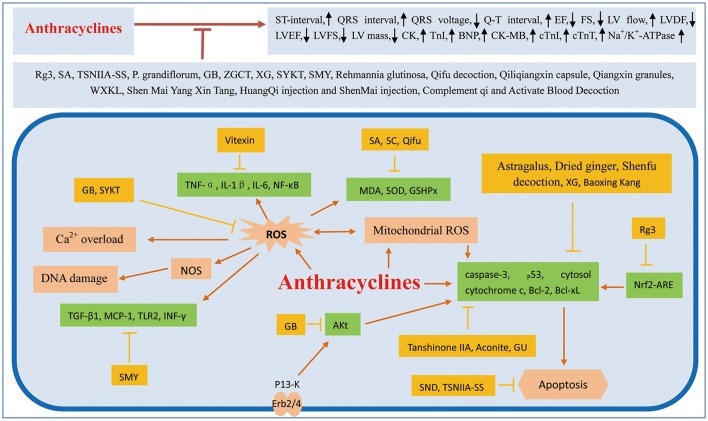
Mechanism involved in traditional Chinese medicine treated on cardiotoxicity induced by anthracycline. ROS, reactive oxygen species; EF, ejection fraction; FS, fractional shortening; LV flow, left ventricular outflow; LVDF, left ventricular diastolic function; LVEF, left ventricle ejection fraction; LVFS, left ventricular fractional shortening; LV mass, left ventricular mass; CK, creatine kinase; Tnl, troponin I; BNP, brain natriuretic peptide; CK-MB, creatine kinase isoenzymes; cTnT, cardiac Troponin T, Rg3, GinsenosideRg3; SA, Salvianolic acids; TSNIIA-SS, Tanshinone IIA sodium sulfonate; P. grandiflorum, *Platycodon grandiflorum*; GB, Ginkgolide B; ZGCT, Zhi Gan Cao Tang; XG, Xinfuli Granule; WXKL, Wenxin Granule; SYKT, Sanyang Xuedai; SMY, Sheng-Mai Yin; NOS, nitric oxide synthase; SND, Sini decoction; GU, *Glycyrrhiza Uralensis*; MDA, malondialdehyde; SC, *Schisandra chinensis*; SOD, superoxide dismutase.

There are many factors associated with the cardiac toxicity induced by chemotherapeutic agents. Some common cardiovascular complications include QT prolongation, HF, hypotension, ischemia, hypertension, pleural effusion, edema, pericardial effusion, bradyarrhythmia, and thromboembolism. It is necessary to take effective measures to prevent these complications. In recent years, several studies have demonstrated that traditional Chinese medicine can prevent the cardiotoxicity of chemotherapeutics. (Figure 1; Table 1).

**Table 1:** Effect of traditional Chinese medicine on cardiotoxicity induced by anthracyclines.

**Cardiotoxicity**	**TCM (Molecular formula)**	**Anthracyclines**	**Type of study**	**Mechanism of action**	**References**
Heart failure	**1. Monomer**				
	Ginsenoside Rg3 (C_42_H_72_O_13_)	Adriamycin	*in vitro, in vivo*	Increased in the EF and FS, and improved the LV outflow. Activation of the Nrf2-ARE pathway and Akt pathway.	Wang et al., 2015
	Tanshinone IIA (C_19_H_18_O_3_)	Doxorubicin	*in vitro*	Decreased the number of cleaved caspase-3 and cytosol cytochrome c, as well as increased BcL-xL expression.	Hong et al., 2012
	Vitexin (C_21_H_20_O_10_)	Doxorubicin	*in vivo*	Increasing the expression levels of IL-1β, IL-6, NF-κB, and TNF-α, decrease caspase-3 activity, suppressed oxidative stress.	Sun et al., 2016
	Ginkgolide B (C_20_H_24_O_10_)	Doxorubicin	*in vitro, in vivo*	Decreased reactive oxygen species, and activating Akt phosphorylation, improved LVEF and reduced LV mass.	Gao et al., 2016
	**2. Active Ingredients**				
	Salvianolic acids	Doxorubicin	*in vitro, in vivo*	Improvement of ECG, and decline of CK. Blocked oxidative stress.	Jiang et al., 2008
	Xinmailong injection	Epirubicin	*in vivo*	Activated the PI3K/Akt signaling pathway, suppressed the Erk1/2 and P38 MAPK signaling pathways.	Li et al., 2016
	**3. Single Herbs**				
	*Glycyrrhiza uralensis*	Doxorubicin	*in vitro*	Cleavage of caspases 9, 3, and 7 decreased.	Choi et al., 2008
	*Platycodon grandiflorum*	Anthracyclines	in clinic	Detection of ECG, LVDF, CK-MB, BNP, and TnI.	Hao et al., 2017
	*Schisandra chinensis*	Adriamycin	*in vivo*	Nucleic acid and protein synthesis were suppressed, MDA was augmented, GSHPx activity and SOD were decreased.	You et al., 2006
	*Rehmannia glutinosa*	Doxorubicin	*in vitro*	Increased in the levels of Bax, p53, phospho-p53, and Bcl-xL.	Li et al., 2015
	**4. Compound**				
	HuangQi injection and ShenMai injection	Anthracyclines	in clinic	Improvement of LVEF, and cTnI decreased.	Li et al., 2013
	Sini decoction	Doxorubicin	*in vitro*	Decreased SOD activity, augmented reactive oxygen species generation, increased MDA formation, decreased release of LDH, activating the PI3K/Akt signaling pathway.	Chen et al., 2013
	Zhi Gan Cao Tang	Anthracyclines	in clinic	The suppression of Na^+^/K^+^-ATPase.	Wu et al., 2015
	Shenmai Yangxin Decoction	Anthracyclines	in clinic	Improvement of LVEF,and cardiac function. cTnl decreased.	Liang et al., 2013
	Complement Qi and Activate Blood	Epirubicin	in clinic	Improvement of LVEF, left ventricular short shaft shorten rate and left ventricular end-diastolic diameter and end systolic diameter.	Yao et al., 2013
	Xinfuli Granule	Doxorubicin	*in vivo*	Improvement of LVEF and LVFS, lower expression of Bax, and higher expression of Bcl-2.	Lu et al., 2016
	Qiangxin Granules	Epirubicin	in clinic	BNP, cTNI, CK-MB decreased. Improvement of LVEF, and ECG.	Zhou and Wang, 2016
	Qiliqiangxin capsule	Cisplatin	in clinic	CK-MB, and cTnT decreased.	Liu et al., 2013
	Sanyang Xuedai	Doxorubicin	*in vivo*	Inhibited reactive oxygen species-mediated apoptosis, increased CD34^+^/CD44^+^ cell counts, and CK-MB decreased.	Chen et al., 2017
	Sheng-Mai Yin	Doxorubicin	*in vivo*	BNP, TGF-β1, and CK-MB decreased. The increase of HWI and LVMI. The inhibition of TLR2, MCP-1, INF-γ, and IL-6.	Ma et al., 2016
Cardiac Arrhythmia	**Compound**				
	Wenxin Granule	Anthracyclines	in clinic	CK, CK-MB, and cTnI decreased.	Lyu et al., 2007; Guohua and Mingmei, 2011
	Qifu Decoction	Adriamycin	*in vivo*	Heart rate increased, QRS voltage increased and Q-T interval shortened, SOD and GSHPx strengthened, and the content of MDA decreased.	Yu et al., 2011
	Shenfu decoction	Adriamycin	*in vivo*	Bcl-2 increased, Bax, Caspase-9, Caspase-3, and cytochrome C decreased.	Sun et al., 2012
	Baoxin Kang	Adriamycin	in clinic	SOD, MDA, and GSHPX decreased.	Sui et al., 2004
Cardiomyopathy	**1. Monomer**				
	Tanshinone IIA sodium sulfonate (C_19_H_17_NaO_6_S)	Doxorubicin	*in vitro, in vivo*	Decreasing ST-interval and QRS interval by ECG, increasing myocardial tensile strength using TTR assay.	Jiang et al., 2009
	**2. Single Herbs**				
	Panax ginseng	Adriamycin	*in vivo*	Bcl-2, Bax, cytc, Caspase-9 and Caspase-3 decreased.	Fan et al., 2011
	Mongholicus				
	Zingiber officinale				
	Aconitum carmichaeli				
	**3. Compound**				
	Sini decoction	Doxorubicin	*in vivo*, in clinic	Partially adjusting the perturbed metabolic pathways.	Tan et al., 2011

*TCM, traditional chinese medicine; ROS, reactive oxygen species; Nrf2-ARE, nuclear factor erythroid 2 related factor-antioxidant response element; EF, ejection fraction; FS, fractional shortening; LV flow, left ventricular outflow; ECG, electrocardiogram; LVDF, left ventricular diastolic function; CK-MB, creatine kinase isoenzymes; BNP, brain natriuretic peptide; TnI, troponin I; TNF-α, tumor necrosis factor-α; GSHPx, glutathione peroxidase; SOD, superoxide dismutase; MDA, malondialdehyde; LVFS, left ventricular fractional shortening; cTnI, cardiac troponin I; TTR, tension to rupture; cTNT, troponin T*.

### Heart failure (HF)

In the clinical or basic experimental, anthracycline-induced plasma brain natriuretic peptide (BNP) levels were significantly higher in patients with left ventricular (LV) dysfunction. Anthracycline-iron chelates have a high affinity for cardiac phospholipids, and when combined with cardiolipin can cause cardiotoxicity in organelle membranes (Nelson et al., 2001; Floyd et al., 2005; Kim et al., 2006). Anthracyclines can inhibit the expression of Ca^2+^-ATPase genes in the myocardial sarcoplasmic reticulum. This would then affect the biosynthesis of Ca^2+^-ATPase leading to a decrease in its activity which would result in reduced Ca^2+^ uptake in the sarcoplasmic reticulum, decreased ATP levels in the mitochondria, decreased myocardial energy metabolism, exacerbated cell damage, and even to myocardial cell death (Trachtenberg et al., 2011). Traditional Chinese medicine can significantly reduce myocardial mitochondrial malondialdehyde (MDA) content, and increase superoxide dismutase (SOD), succinodehydrogenase (SDH), and Ca^2+^-ATPase activity (Jin and Sun, 2006). Additionally, traditional Chinese medicine can effectively prevent free radicals produced by anthracycline from damaging normal cardiomyocytes, without affecting its anti-tumor effect. In this section, we summarized the protective effect of traditional Chinese medicine on anthracycline-induced HF.

#### GinsenosideRg3

Ginseng is well-known in herbal medicine as a restorative and tonic agent. Ginsenoside Rg3 (Rg3), an active ingredient isolated from panax ginseng, is used to prevent and inhibit cancers (Lu et al., 2008). Rg3, an anti-cancer agent, was reported to possess anti-oxidative (Shin et al., 2013), anti-apoptotic, and cardioprotective properties (Lim et al., 2013). Wang et al. (2015) investigated the cardioprotective effect of Rg3 on adriamycin-treated rats. Cardiac endothelial cells were incubated to investigate the role of Rg3 on adriamycin-treated cells (Wang et al., 2010). Results indicated that Rg3 could improve the ejection fraction (EF) and fractional shortening (FS), induced by adriamycin, and improve the LV outflow. The aortic ring assay revealed that Rg3 could partly recuperate the unusual vascular function. Studies have indicated that Rg3 could facilitate the viability of cells to attenuate adriamycin-induced oxidative injury and apoptosis, by activating Akt which then activates the nuclear factor erythroid 2 related factor-antioxidant response element (Nrf2-ARE) pathway (Li et al., 2000; Wang et al., 2015).

#### Tanshinone IIA

Tanshinone IIA is extracted from Danshen, a prevalent medicinal herb used in traditional Chinese medicine. It possesses vasodilatory and cardioprotective properties (Zhou et al., 2005; Gao et al., 2008; Sun et al., 2008; Xu et al., 2009). Hong et al. (2012) employed primary cultured rat cardiomyocytes to assess the protective role of tanshinone IIA on doxorubicin-induced cardiac myocyte apoptosis. The result showed that tanshinone IIA limited doxorubicin-induced reactive oxygen species production, decreased the number of cleaved caspase-3 and cytosol cytochrome c, and increased B-Cell Lymphoma-Extra Large (Bcl-xL) expression, thereby protecting cardiomyocytes from doxorubicin-induced apoptosis. Furthermore, Akt phosphorylation was reinforced by tanshinone IIA treatment in the cardiac myocytes (Jin et al., 2008). The transfection of Akt small interfering ribonucleic acid (siRNA), LY294002, and wortmannin dramatically reduced tanshinone IIA-induced protective action. These findings suggest that tanshinone IIA prevented cardiomyocytes from doxorubicin-induced apoptosis through the Akt-signaling pathway (Fu et al., 2007; Gao et al., 2008; Hong et al., 2012).

#### Vitexin

Hawthorn, of the Rosaceae plant family, is a traditional Chinese medicine that is used to facilitate digestion (Zick et al., 2009). It had been reported that the ketone compounds extracted from hawthorn leaves altered the blood pressure and blood lipid levels, increased coronary flow, and preserved ischemic myocardium (Tassell et al., 2010; Je et al., 2014; Wang X. S. et al., 2014). A previous study demonstrated that vitexin exhibited protective action against hypoxia during reoxygenation in cardiomyocytes (Xue et al., 2014). Sun et al. (2016) investigated whether vitexin could protect against doxorubicin-induced cardiotoxicity in a rat model. The levels of lactate dehydrogenase (LDH), creatine kinase isoenzymes (CK-MB), MDA, SOD, catalase, and myeloperoxidase were evaluated using assay kits. The levels of inflammatory mediators, including tumor necrosis factor-α (TNF-α), interleukin (IL)-1β, IL-6, nuclear factor (NF)-κB, and caspase-3 (Sharma and Das, 1997), were assayed by the enzyme-linked immunosorbent assay method. Western blot analysis was performed to evaluate the protein expression levels of p-FOXO3a (Kim et al., 2014). Vitexin pretreatment dramatically protected against doxorubicin-induced myocardial injury, which was characterized by augmented LDH and serum CK-MB. Vitexin observably reduced oxidative stress injury caused by doxorubicin by inhibiting lipid peroxidation and elevating antioxidant enzyme activities.

#### Ginkgolide B

Ginkgolide B is the main terpenoid ingredient extracted from G. Biloba leaves. Researchers have discovered that ginkgolide b exerts regulation or protective actions by decreasing oxidative stress and Aβ-induced functional disorder of mitochondrial oxidative phosphorylation of the neuronal cells and sustaining cellular energy demands (Kaur et al., 2015). Gao et al. (2016) evaluated the effect of ginkgolide b on doxorubicin-induced cardiotoxicity *in vitro* and *in vivo*. The cardiomyocyte cell line H9c2 was pretreated with ginkgolide b and subsequently subjected to doxorubicin treatment. Cell viability and apoptosis were detected by 3-(4,5-dimethyl-2-thiazolyl)-2,5-diphenyl-2-H-tetrazolium bromide (MTT) assay and Hoechst staining, respectively. Reactive oxygen species, Akt phosphorylation, and intracellular calcium levels were equally determined so as to explore the underlying molecular mechanism. The study established a mouse model of cardiotoxicity and measured LV mass and LVEF (Guenancia et al., 2015; He et al., 2016). The outcome of the *in vitro* experiment indicated that pretreatment with ginkgolide b remarkably reduced the viability and apoptosis of H9c2 cells by decreasing reactive oxygen species, intracellular calcium levels, and activating Akt phosphorylation (Swain et al., 2003; Chen et al., 2013; Cao et al., 2014). In the *in vivo* experiment, they observed an elevated LVEF and a reduced LV mass in the group of cardiotoxic rats treated with ginkgolide b. In conclusion, these findings indicated that combination of ginkgolide b and doxorubicin in chemotherapy could help avert the toxic effects and side effects of doxorubicin.

#### Salvianolic acids

*Salvia miltiorrhiza* is extensively used in China to treat cardiovascular diseases (Zhou et al., 2005). Salvianolic acids, the primary bioactive component of *S. miltiorrhiza*, includes salvianolic acid A, rosmarinic acid, salvianolic acid B, and other phenolic acids (Adams et al., 2006). Salvianolic acids are known to be powerful antioxidative agents that reduce the level of hydroxyl radicals and lipid peroxidation (Wang et al., 2007). Jiang et al. (2008) studied the cardioprotective action of salvianolic acids on acute cardiotoxicity induced by doxorubicin in mice. The study established an acute cardiac damage mouse model. Therapeutic treatment with salvianolic acids decreased doxorubicin-induced toxicity, increased the body weight, heart weight/tibia length ratio, and heart vacuolation, and decreased CK levels. Furthermore, the antioxidative role of salvianolic acids were validated by MDA detection and oxygen radical absorbance capacity (ORAC) assays *in vivo* (Takemura and Fujiwara, 2007; Jiang et al., 2008). This reveals that the blocking of oxidative stress by salvianolic acids is one potential mechanism underlying its role in cardioprotection.

#### Xinmailong injection

Xinmailong, a bioactive compound extracted from *Periplaneta americana*, exhibited reasonable protective properties against cardiovascular damage and was approved for use in therapy for cardiac dysfunction in 2006. In a previous study (Li et al., 2016), rats were intraperitoneally injected with epirubicin and subsequently treated with xinmailong for 14 days. The survival rate, Masson staining, and ECG were used to assess the cardioprotective activity of xinmailong (Bhandare et al., 2015; Prysyazhna et al., 2016). Quantitative real time reverse transcriptase polymerase chain reaction (RT-PCR) analyses and western blotting were used to study molecular mechanisms underlying the effects of xinmailong. They showed that xinmailong was efficient in activating the PI3K/Akt signaling pathway and suppressing the Erk1/2 and P38 MAPK signaling pathways. Xinmailong prevented left ventricle dilatation and improved cardiac function (Liu et al., 2014; Wang X. et al., 2014; Li et al., 2016). Additionally, xinmailong treatment dramatically enhanced the survival rate of rats with epirubicin-induced HF.

#### Hexane/ethanol extract of *Glycyrrhiza uralensis*

Licorice is one of the constantly prescribed agents in traditional herbal medicine, and is employed as a natural sweetening additive. In traditional Chinese medicine, the licorice root is added to all kinds of herbal preparations to detoxify other herbs. Choi et al. (2008) explored the possibility that the hexane/ethanol extract of *Glycyrrhiza uralensis* may relieve doxorubicin-induced cardiotoxicity. H9c2 cells were pretreated with the hexane/ethanol extract of *G. uralensis*, and then treated with doxorubicin. The pretreatment led to a significant alleviation of doxorubicin-induced decreases in cells and reduced apoptosis. The western blot analysis of cell lysates revealed that the hexane/ethanol extract of *G. uralensis* inhibited doxorubicin-induced increase in the levels of Bax, p53, and phospho-p53 (Levine, 1997; Shieh et al., 1997; Chehab et al., 1999; Choi et al., 2008). Additionally, the hexane/ethanol extract of *G. uralensis* induced an increase in the levels of Bcl-xL (Häcker and Weber, 2007; Lalier et al., 2007). Pre-treatment with the hexane/ethanol extract of *G. uralensis* suppressed the doxorubicin-induced cleavage of caspases 9, 3, and 7, and doxorubicin-induced polymerase cleavage. These results indicated that the hexane/ethanol extract of *G. uralensis* may potentially be an efficient agent for the mitigation of doxorubicin-induced cardiotoxicity.

#### Platycodon grandiflorum

*Platycodon grandiflorum* is a perennial flowering plant of the Campanulaceae family and the only species of the genus Platycodon. It is an herbal medicine that has been used in traditional Chinese medicine for thousands of years to treat cardiovascular diseases (Oh et al., 2010; Bae et al., 2011; Zhang et al., 2015). In traditional Chinese medicine theory, *P.grandiflorum* can nourish Qi and remit symptoms, such as chest pain, palpitations, and shortness of breath. One study (Hao et al., 2017) assessed the cardioprotective actions and safety of *P.grandiflorum* granules in patients with early breast cancer who were receiving anthracycline-based chemotherapy. A total of 120 patients randomly received either *P. grandiflorum* granules or placebo granules. The main outcome was HF, as well as the secondary outcomes contained all-cause mortality, ECG, LV diastolic function (LVDF), cardiac death, longitudinal systolic strain, and velocities measured by tissue Doppler imaging, cardiac biomarkers, such as CK-MB, brain natriuretic peptide (BNP), and troponin I (TnI) (Murtagh et al., 2016). The clinical trial evaluated the cardioprotective role and safety of *P.grandiflorum* in patients with early breast cancer receiving anthracycline-based chemotherapy.

#### Schisandra chinensis

A classical treatise on Chinese herbal medicine, written by Shen Nung Pen Tsao Ching, described *Schisandra chinensis* or Wu Wei Zi as a high-grade herb which is useful for treating a wide variety of medical conditions. It has strong antioxidative properties (Ichikawa and Konishi, 2002). You et al. (2006) surgically removed the hearts of rats and measured the rate of synthesis of proteins and nucleic acids. They also measured the rate of lipid peroxidation and the level of myocardial antioxidants. Their results revealed that nucleic acid and protein synthesis was suppressed while MDA levels increased. However, myocardial glutathione peroxidase (GSHPx) activity and SOD were decreased by adriamycin (Morishima et al., 1998). In contrast, administration of *S.chinensis* before and concurrently with adriamycin dramatically reduced ascites and mortality. Indices of myocardial GSHPx, SOD activities, and macromolecular biosynthesis increased with a concomitant reduction in lipid peroxidation (Ko et al., 1995; Guenancia et al., 2015). Adriamycin-induced cardiotoxicity was associated with antioxidant deficits and *S.chinensis* treatment could alter antioxidant levels in the heart and ameliorate cardiac function.

#### Rehmannia glutinosa

*Rehmannia glutinosa*, a member of the Scrophulariaceae family, has been used in traditional Chinese herbal medicine for thousands of years. It was recorded in the Chinese medical classic “Shennong's Herba” and was considered as a “top grade” herb in China (Li et al., 2015). In recent studies, a new ionone glycoside, frehmaglutoside I (1), and three new rhemaneolignans A–C (2–4) were extracted from the 95% EtOH isolate from the roots of *R. glutinosa* (Zhang et al., 2013; Feng et al., 2015). Their structures were confirmed by extensive spectroscopic analyses. These compounds were assessed for their protective actions against cardiocytes damaged by treatment with doxorubicin. Compounds 1–3 exhibited protective roles against doxorubicin-induced cardiotoxicity.

#### HuangQi injection and ShenMai injection

Most Chinese herbal medicines can enhance the scavenging of endogenous active oxygen free radicals, prevent myocardial ischemia and arrhythmia, and reduce lipid peroxidation (Liu et al., 2012; Cai et al., 2013). HuangQi was mainly extracted from radix astragali, and can regulate the balance of free radicals in the body, thus protecting the heart (Yang et al., 2001). ShenMai injection consisted of radix ginseng and radix ophiopogonis. Clinical research confirmed that ShenMai injection could remarkably prevent cardiac injury induced by chemotherapy (Yang et al., 2012). One hundred and twenty patients with malignant tumors were randomly assigned to four groups (groups A, B, C, and D). All the patients were treated with chemotherapy regimens including anthracyclines (Junjing et al., 2010; Li et al., 2013). Patients in groups A, B, C, and D received HuangQi injection, ShenMai injection, deferoxamine, and traditional Chinese medicine injection combined with deferoxamine, respectively, for four chemotherapy cycles based on the original chemotherapy regimens. A cycle comprised 28 days. Changes in ECG, LVEF, and qualitative detection of cardiac TnI (cTnI) were monitored before and after treatment. The study demonstrated that HuangQi and ShenMai injections had certain protective effects against the cardiac toxicity induced by anthracyclines (Junjing et al., 2010).

#### Sini decoction

Sini decoction is a renowned formula in traditional Chinese medicine, and was officially recorded in the Chinese Pharmacopoeia, 2010 Edition. It comprises three medicinal herbs: aconiti lateralis radix praeparata, *Glycyrrhiza uralensis*, and *Rhizoma zingiberis*. Several studies have shown that sini decoction is an efficient agent in the treatment of cardiovascular diseases (Wu et al., 1999; Jin et al., 2003; Liang, 2005). A recent study (Chen et al., 2013) investigated the active components in sini decoction and their cardioprotective actions in doxorubicin-induced cytotoxicity. The results demonstrated that treatment with higenamine or [6]-gingerol increased the viability of doxorubicin-injured cardiomyocytes. Moreover, the study also found that treatment with doxorubicin decreased SOD activity, augmented reactive oxygen species generation, increased MDA formation, decreased the release of LDH, and activated the mitochondria-dependent apoptotic pathway in cardiomyocytes (Kaiserová et al., 2006; Li et al., 2006; Danz et al., 2009; Zhang S. et al., 2012). These effects of treatment with doxorubicin were suppressed by co-treatment with higenamine and [6]-gingerol. Most importantly, the cytoprotection provided by higenamine and [6]-gingerol could be eliminated by LY294002, a PI3K inhibitor. A combination of higenamine and [6]-gingerol plays a cardioprotective role against doxorubicin-induced cardiotoxicity by activating the PI3K/Akt signaling pathway (Ha et al., 2012), and higenamine and [6]-gingerol may be the active ingredients in sini decoction. Thus, these results demonstrated the protective role of sini decoction in doxorubicin-induced HF (Zhao et al., 2005, 2009; Chen et al., 2013).

#### Zhi Gan Cao Tang

Zhi Gan Cao Tang is an old Chinese herbal medicine formula composed of nine raw herbal components: radix glycyrrhizae, radix ginseng, radix ophiopogonis, fructus jujubae, radix rehmanniae, colla corii asini, ramulus cinnamomi, fructus cannalis, and rhizoma zingiberis recens. Ginsenoside and glycyrrhizin isolated from radix glycyrrhizae and radixginseng, respectively, are steroid-like chemical compounds which are structurally analogous to cardenolides such as digitoxin and digoxin (Chen et al., 2011). These steroid-like chemical compounds have therapeutic roles and are used for treating HF via the identical molecular mechanism triggered by the suppression of Na^+^/K^+^-ATPase (Chen et al., 2011). In an interesting case involving an adolescent male with refractory acute lymphoblasticleukemia (ALL) who had suffered from anthracycline-induced congestive HF (Wu et al., 2015), this patient who previously could not perform daily activities and relied on supplemental oxygen, could perform these activities without supplemental oxygen after 6 days of treatment with traditional Chinese medicine. After Chinese herbal medicine treatment for 2 months, the follow-up chest X-ray indicated improvements in cardiomegaly and pulmonary edema. In this circumstance, anthracycline-induced cardiotoxicity was gradually resolved following the oral administration of Zhi Gan Cao Tang.

#### Shenmai Yangxin decoction

Shen Mai Yang Xin Tang contains astragalus, ginseng, and ophiopogon. It can eliminate oxygen free radicals, and promote the recovery of cardiac function (Cao et al., 2010). Liang et al. (2013) studied 62 patients with breast cancer and randomly separated them into two groups: the treatment and control groups. The two groups received the same chemotherapy regimen, symptomatic support, and western medicine treatment. The incidence of cardiotoxicity-related symptoms including changes in cardiac function, ECG, LVEF, and cTnI were noted before and after chemotherapy. The incidence of chest discomfort and palpitations were 16.7 and 13.9% in the treatment group, respectively, and 46 and 42.3% in the control group, respectively. There was no significant dyspnea in both groups. The cardiac function of the treatment group was significantly improved compared to that of the control group. The rate of abnormalities in the ECG was 25 and 11.1% in the treatment group, and 57.6% and 34.6% in the control group. After chemotherapy, cTnI increased significantly in the treatment group (Ganame et al., 2007; Liang et al., 2013), but was only slightly higher in the treatment group after intervention with traditional Chinese medicine. These results suggested that intervention with shenmai yangxin decoction can reduce drug toxicity and enhance tolerance to anthracyclines in patients.

#### Complement Qi and activate blood decoction

The main components of complement qi and activate blood decoction are ginseng, astragalus, zhigancao, ophiopogon japonicus, schisandra, angelica, salvia, chuanxiong, and guizhi. A recent study (Yao et al., 2013) showed the clinically curative effect of complement qi and the effect of activate blood circulation method in preventing and controlling cardiac toxicity caused by epirubicin. Hundred patients undergoing chemotherapy with a regimen containing epirubicin were randomly divided into treatment and control groups. Changes in ECG and color doppler ECG before and after the treatment were noted. The incidence of abnormal ECG in the treatment group was lower than that in the control group. The LVEF, stroke index, LV short shaft shorten rate, and LV end-diastolic and end systolic diameters of the two groups were improved after chemotherapy. These results showed that the complement qi and activate blood circulation method can effectively control cardiac toxicity caused by epirubicin (CaI and Li, 2008; Gu et al., 2008; Li et al., 2008; Zhang and Ji, 2011; Yao et al., 2013).

#### Xinfuli granule

The xinfuli granule is a compound traditional Chinese medicine consisting of extracts from radix astragali, radix ginseng, *S. miltiorrhiza, Scirpus fluviatilis*, rhizoma alismatis, *Angelica sinensis*, semen lepidii, fructus chaenomelis, semen arecae, and *Ophiopogon japonicus* formulation. It has been clinically used for the treatment of HF for over 50 years (Kang and Izumo, 2000; Abbate et al., 2003). Recently, a study (Lu et al., 2016) showed the effects and mechanisms of xinfuli in rats with doxorubicin-induced cardiotoxicity. Rats were treated with an intraperitoneal injection of doxorubicin for 6 weeks. They were then randomly assigned to four groups. Each group received intragastric administration of either normal saline or different dosages of xinfuli granule for 6 weeks. Transthoracic echocardiography was conducted to evaluate the LVEF and LVFS before and after the xinfuli granule treatment. Myocardial cell apoptosis was assessed using TUNEL staining. The expression of associated genes and proteins were analyzed by employing immunohistochemical staining. Compared with the control group, xinfuli granule treated groups showed significantly ameliorated cardiac function (Ling et al., 2009), cardiac histopathological changes, lower cardiomyocyte apoptosis index (Tao et al., 2015), lower expression of Bax, and higher expression of Bcl-2. These results revealed that administration of xinfuli granule improved histopathological and cardiac function variations in rats with doxorubicin-induced cardiotoxicity (Lu et al., 2016).

#### Qiangxin granules

Qiangxin granules are obtained from fangmu fangzao decoction and are composed of ginseng, gui zhi, salvia, ting lizi, han defense, motherwort, and citrus aurantium. Clinically, it was used for the treatment of patients with HF (Zhang H. X. et al., 2012). A study (Zhou and Wang, 2016) was conducted involving 60 patients with breast cancer, lung cancer, liver cancer, leukemia, and lymphoma. They were randomly separated into a treatment group and control group, with 30 cases in each group. The treatment group was treated with traditional Chinese medicine (qiangxin granules) instead of the original chemotherapy regimen, while the control group continued to use the original chemotherapy regimen. After 4 weeks, the changes in LVEF, QTc interval, plasma BNP, cTnl, and CK-MB were measured in both groups. After qiangxin granule treatment, the expression of LVEF, BNP, cTNI, CK-MB, and the ECG QTc interval were significantly different between groups (Li et al., 2009; Fazlinezhad et al., 2011). These results revealed that qiangxin granules had a significant effect on epirubicin-induced cardiotoxicity.

#### Qiliqiangxin capsule

The qiliqiangxin capsule is mainly composed of astragalus, ginseng, aconite, salvia, tinglizi, alisma, polygonatum, guizhi, safflower, fragrant skin, and dried tangerine peel, and provides myocardial protection (Zou et al., 2012). Nineteen patients with cancer were enrolled in the study and randomly divided into treatment and control groups (Liu et al., 2013). In the treatment group, the patients received qiliqiangxin capsule combined with radiotherapy. The qiliqiangxin capsule was administered orally a day before the commencement of radiotherapy. In the control group, the patients received the standard dose of chemotherapy. Three weeks was regarded as one cycle in the two groups. Serum levels of myocardial markers CK-MB and cardiac troponin T (cTnT) were measured in all patients before and after each chemotherapy cycle. CK-MB and cTnT levels were found to be statistically different between the two groups after chemotherapy. The qiliqiangxin capsule can inhibit cardiotoxicity induced by recombinant human endostatin combined with radiotherapy (Zou et al., 2012; Liu et al., 2013).

#### Sanyang xuedai

Sanyang xuedai is a natural medicine from an ancient herbal formula of the Dai nationality in Southwest China. With eight herbal medicines, including sanguis draconis, radix etrhizoma glycyrrhizae, radix etrhizoma notoginseng, and radix angelicae sinensis as the main ingredients, sanyang xuedai has been reported to promote many biological functions (Lv et al., 2004; Zhou et al., 2015). Chen et al. (2017) investigated whether sanyang xuedai can protect against doxorubicin-induced cardiotoxicity and myelosuppression. Mice were treated with doxorubicin, sanyang xuedai, or a combination of both. Hematopoietic functions were evaluated by measuring clusters of differentiated CD34^+^/CD44^+^ bone marrow cells, number of peripheral blood cells, and apoptotic cells. Myocardial enzymes, embracing CK, aspartate aminotransferase, LDH, and its isoform CK-MB, were evaluated by using a biochemical analyzer. The rate of apoptosis in cardiac myocytes was assessed using flow cytometry. Histopathological analysis was performed by hematoxylin and eosin (H&E) staining. Intracellular reactive oxygen species generation was assessed using a dichlorofluorescein intensity assay. Mice treated with doxorubicin exhibited a decreased survival rate, decreased peripheral blood and CD34^+^/CD44^+^ cell counts, and increased myocardial enzymes and apoptotic indices, all of which were efficiently prevented by sanyang xuedai co-administration (Kluza et al., 2004; Chen et al., 2017). In addition, bone marrow cells and myocytes from mice treated with doxorubicin exhibited increased dichlorofluorescein intensity, which was weakened by sanyang xuedai (Lv et al., 2004; Chen et al., 2017). The present study demonstrated that sanyang xuedai may offset doxorubicin-induced myelosuppression and cardiotoxicity by inhibiting reactive oxygen species-mediated apoptosis.

#### Sheng-Mai Yin

Sheng-Mai Yin, a traditional Chinese formula diffusely used for coronary heart disease treatment with “qi–yin” deficiency, constitutes three herbal medicines, namely radix ginseng, radix ophiopogonis, and fructus schisandrae (Ma et al., 2003). It is based on traditional Chinese medicine theory, and has been employed to treat cardiovascular diseases in Asia. Ma et al. (2016) studied the cardioprotective role of Sheng-Mai Yin against doxorubicin-induced cardiac toxicity *in vivo*. Rats were injected with doxorubicin six times over a period of 2 weeks. Sheng-Mai Yin was administrated intragastrically together with doxorubicin for this period. A train of assays were performed to test the effects of Sheng-Mai Yin on heart weight index (HWI), LV mass, cardiac function, heart tissue morphology, transforming growth factor-β1 (TGF-β1), BNP, monocyte chemoattractant protein-1 (MCP-1), interferon gamma (INF-γ) and IL-6, mRNA levels of TGF-β1, toll-like receptor-2 (TLR2), and protein levels of TGF-β1. The results showed that rats treated with Sheng-Mai Yin exhibited reductions in BNP levels. The CK-MB levels were increased by doxorubicin in a dose-dependent way. Moderate doses of Sheng-Mai Yin corrected the increased HWI and LVMI, injured cardiac function, as well as collagen accumulation (Horenstein et al., 2000; Migrino et al., 2008; Geisberg and Sawyer, 2010; Psaltis et al., 2011; Zhou et al., 2014). Furthermore, the cardioprotective action of Sheng-Mai Yin against doxorubicin-induced cardiac toxicity was demonstrated by the reduction in myocardial fibrosis.

### Cardiac arrhythmia

Anthracyclines can activate the sarcoplasmic reticulum Ca^2+^ channel, increasing the release of sarcoplasmic reticulum Ca^2+^ (RVitelli et al., 2002). High levels of free Ca^2+^ intracellularly can cause the rapid increase in cardiac activity resulting in a variety of arrhythmias. Traditional Chinese medicine can reduce cell calcium concentration, intracellular free calcium, and changes in activity, thereby mitigating anthracycline myocardial toxicity. In the preceding text, we reviewed the protective effect of traditional Chinese medicine on anthracycline-induced cardiac arrhythmia.

#### Wenxin granule

Wenxin granule is clinically effective in the treatment of arrhythmia compared to drugs like Codonopsis, Polygonatum, Nansong, Panax, and Amber. Some studies demonstrated that wenxin granules are superior to amiodarone for the treatment of patients with arrhythmia caused by anthracycline antineoplastic drugs (Lyu et al., 2007; Aimin, 2011; Guohua and Mingmei, 2011). The use of wenxin granules has a few side effects which are less recurrent. Two studies (Lyu et al., 2007; Guohua and Mingmei, 2011) evaluated the effect of wenxin granules on arrhythmia caused by anthracycline chemotherapy. Patients with confirmed malignant tumors were randomly divided into treatment and chemotherapy groups. The patients received anthracycline antitumor drugs combined with systemic chemotherapy and wenxin granule three times a day. The result showed that wenxin granules can reduce arrhythmic toxicity caused by anthracycline chemotherapy, and is applicable clinically.

#### Qifu decoction

Adriamycin is a widely used broad-spectrum anti-tumor drug in clinical settings, but can lead to cumulative cardiotoxicity. The affinity of adriamycin for the myocardium is significantly higher than that for other tissues. In the myocardium, adriamycin can cause toxic effects (Quiles et al., 2002), early cardiac arrhythmia, mainly during sinus tachycardia, QRS low voltage, and Q-T interval prolongation. Yu et al. (2011) investigated the effect of qifu decoction on heart function in adriamycin-induced cardiac injury in a rat model. Heart function was measured using ECG. The activities of SOD and GSHPx were detected, and the content of the lipid peroxidation product MDA was assessed by the chemical colorimetric method. The results showed that the heart rate of rats in the model group slowed down, the sum of QRS voltage decreased, and the Q-T interval increased (Büyükokuroglu et al., 2004). Compared to the model group, the heart rate of rats in the qifu decoction group increased, the sum of QRS voltage increased, and the Q-T interval shortened. In addition, the activities of SOD and GSHPx strengthened, and the content of the MDA decreased in the qifu decoction group (Li and Li, 2009; Wang et al., 2009). Qifu decoction can prevent adriamycin-induced cardiotoxicity in the rat model, and its underlying mechanism may be associated with antioxidant stress.

#### Shenfu decoction

There is a close relationship between adriamycin-induced cardiac injury and the mitochondrion signaling pathway of cardiomyocyte apoptosis (Green and Leeuwenburgh, 2002; Chae et al., 2005; Fisher et al., 2005; Oliveira et al., 2006). A recent study (Sun et al., 2012) demonstrated the effect of shenfu decoction on the mitochondrion signaling pathway during adriamycin-induced cardiomyocyte apoptosis in a rat model. Enzyme-linked immunosorbent assay (ELISA) was used to measure the levels of Caspase-9, Caspase-3, Bcl-2, Bax, and cytochrome C. The results demonstrated that the level of Bcl-2 decreased in the adriamycin-induced cardiac injury rat model (Hanada et al., 1995; Adams and Cory, 1998; Sun et al., 2012). However, the levels of Bax, Caspase-9, Caspase-3, and cytochrome C increased. Shenfu decoction can protect cardiomyocytes from adriamycin-induced cardiac injury by inhibiting cardiomyocyte apoptosis in the mitochondrion signaling pathway.

#### Baoxin kang

Baoxing kang is composed of ginseng, astragalus, and epimedium as primary components, and it is used to treat congestive HF (Chen, 1996). Sui et al. (2004) investigated the effect of baoxin kang on adriamycin cardiotoxicity. Hundred patients with malignant tumor were divided into a prevention group (60 patients) and control group (40 patients), 10 days before adriamycin chemotherapy with oral administration of the traditional Chinese medicine baoxin kang. The control group received traditional oral anti-oxidized western medicine, Vitamin C, Vitamin E, and Coenzyme QI0. In the adriamycin chemotherapy day plus a lot of vitamin C intravenous infusion once. The two groups were continuously medicated for 5 weeks during the course of treatment. The activities of SOD, MDA, and GSHPX in the serum were measured before and after chemotherapy on days 1, 7, 14, and 28. The incidence of cardiotoxicity was assessed in both groups. The results revealed that Baoxin Kang had a significant preventive effect on the cardiotoxicity induced by doxorubicin (Wang et al., 1994; Sui et al., 2004), and its preventive effect is superior to that of western antioxidant and cardioprotective agents.

### Cardiomyopathy

Anthracyclines can cause a reduction in myocardial antioxidant enzymes such as superoxide dismutase and glutathione peroxidase. This can subsequently prevent the prompt removal of free radicals and superoxide, resulting in aggravated cardiomyocyte damage (Keefe, 2001; Carvalho et al., 2014). Traditional Chinese medicine can significantly reduce anthracycline-induced myocardial injury, increase myocardial SOD activity, and increase oxygen free radical scavenging, thereby reducing the quantity of oxygen free radicals during myocardial injury (Wang and Wu, 2004). In this segment, we su mmed up the protective effect of traditional Chinese medicine on anthracycline-induced cardiomyopathy.

#### Tanshinone IIA sodium sulfonate

Tanshinone IIA sodium sulfonate, a water-soluble derivative of tanshinone IIA, has been widely used as a traditional Chinese medicine for patients with cardiovascular diseases (Ji et al., 2000). A study (Jiang et al., 2009) showed that tanshinone IIA sodium sulfonate exhibits a protective effect against doxorubicin-induced cardiotoxicity. *In vitro* research on H9c2 cell line, as well as *in vivo* investigation in animal models of the doxorubicin-induced chronic cardiomyopathy have been conducted. By using a CCK-8 assay and Hoechst 33342 stain it was demonstrated that tanshinone IIA sodium sulfonate significantly augmented cell viability and improved apoptosis of doxorubicin-injured H9c2 cells. In addition, the cardioprotective actions of Tanshinone IIA sodium sulfonate were verified from the decreasing ST-interval and QRS interval in the ECG (Jiang et al., 2008). The improved appearance of the myocardium in the H&E stain, increased myocardial tensile strength measured using the tension to rupture (TTR) assay, and the reduced fibrosis revealed by the picric-sirius red staining also confirmed the cardioprotective action of tanshinone IIA (Takahashi et al., 2002; Jiang et al., 2009). These studies provided considerable evidence that tanshinone IIA sodium sulfonate is a protective agent against doxorubicin-induced cardiac injury.

#### Panax ginseng, astragalus, dried ginger, aconite

Panax ginseng, astragalus, dried ginger, and aconite play a role in preventing myocardial cell damage, thus improving cardiac function (Lei and Wang, 1993). Fan et al. (2011) studied the effects of ginseng, astragalus, aconite, and ginger on mitochondrial pathway apoptosis in rats with adriamycin-induced cardiac toxicity. The levels of Bcl-2, Bax, cytc, Caspase-9, and Caspase-3 in the myocardium were measured by ELISA (Adams and Cory, 1998). Adriamycin-induced cardiotoxicity induced mitochondrial pathway cell apoptosis in rats. However, panax ginseng, astragalus, dried ginger, and aconite regulated the mitochondrial pathway of apoptosis to promote myocardial function.

#### Sini decoction

Sini decoction, which is officially recorded in the Chinese Pharmacopoeia, 2010 edition and has been employed to prevent or treat cardiovascular disease for many years (Wu et al., 2001; Jin et al., 2003; Zhao et al., 2008, 2009), is a typical traditional Chinese medicine. It is composed of three medicinal plants, including zingiber officinale, acontium carmichaeli, and glycyrrhiza uralensis. A tissue-targeted metabonomic method employing gas chromatography mass spectrometry was used to characterize the metabolic profile of the doxorubicin-induced cardiomyopathy in mice (Tan et al., 2011). Using Elastic Net for classification and election of biomarkers, 24 metabolites correlated with doxorubicin-induced cardiomyopathy were filtered out. These metabolites are primarily implicated in glycolysis, the citrate cycle, lipid metabolism, and some amino acid metabolism (Tokarska-Schlattner et al., 2006; Schulz et al., 2010). With these altered metabolic pathways as possible drug targets, the protective action of the traditional Chinese medicine sini decoction was systematically analyzed. It was reported that sini decoction administration prevented doxorubicin-induced cardiomyopathy by partially adjusting the perturbed metabolic pathways (Zhao et al., 2009; Tan et al., 2011). In conclusion, sini decoction can effectively relieve cardiotoxic response, and decrease the degree of myocardial damage (Xu, 2017).

## Conclusions

In summary, the mechanism underlying the anthracycline-induced cardiotoxicity is very complicated, and may be a multi-gene, multi-factor, and multi-step process. Anthracyclines can cause oxidative stress and free radical formation, calcium overload, apoptosis, mitochondrial damage, and the interaction between multiple factors, leading to the occurrence and development of cardiac toxicity. Traditional Chinese medicine can increase the level of GPX and SOD, reduce the formation of oxygen free radicals, improve anti-lipid peroxidation damage, and decrease NO production and calcium antagonism. This results in the reduction of the concentration of Ca^2+^ in cardiomyocytes, leading to the inhibition of the apoptosis of cardiomyocytes. By so doing, the activity of Na^+^/K^+^-ATPase is enhanced in the myocardium and both mitochondrial membrane potential and GPX activity are increased. Traditional Chinese medicine can also significantly reduce the cardiac toxicity of anthracyclines, protect heart function, and improve the quality of life. Additionally, it can ensure the smooth progress of chemotherapy which maximizes patient treatment and reduces treatment-related mortality. In addition, most of the traditional Chinese medicines have anti-cancer effects, with few side effects. Therefore, traditional Chinese medicine has attracted increasing attention.

## Author contributions

HS and YX defined the research theme. YT, NL, XL, HT, and XW searched for related articles. HT and YT revised and proofreaded the manuscript. LL, LJ and YG collated all related articles. YY wrote the manuscript. All authors commented on the manuscript.

### Conflict of interest statement

The authors declare that the research was conducted in the absence of any commercial or financial relationships that could be construed as a potential conflict of interest. The reviewer JC declared a shared affiliation, though no other collaboration, with several of the authors XY, XL, YY, XW, YG, HS to the handling Editor.
